# Investigating endogenous pain modulation through pupillometry: can pupil size reflect offset analgesia?

**DOI:** 10.1097/PR9.0000000000001507

**Published:** 2026-10-13

**Authors:** Anna Leonhartsberger, Iwanka Iwanojko, Joëlle Katharina Metzger, Florin Allmendinger, Michèle Hubli

**Affiliations:** a Spinal Cord Injury Center, Balgrist University Hospital, University of Zurich, Zurich, Switzerland; b Neuroscience Center Zurich, University of Zurich and ETH Zurich, Zurich, Switzerland

**Keywords:** Offset analgesia, Pupillometry, Descending control, Endogenous pain modulation, Pupillary reaction, Autonomic nervous system

## Abstract

**Introduction:**

Painful stimuli trigger autonomic nervous system responses, including changes in pupil size, which provide a physiological measure of pain processing and modulation. The endogenous pain modulatory system plays a key role in regulating pain, and its dysfunction contributes to a variety of chronic pain conditions. Offset analgesia (OA) offers a behavioral measure of this system but relies on subjective ratings.

**Objectives:**

This study aimed to determine whether pupil size can serve as a physiological proxy of OA in healthy individuals, complementing traditional pain ratings.

**Methods:**

Twenty-nine healthy participants completed an OA paradigm applied on the left volar forearm with 3 thermal phases: T1, 47°C for 5 seconds; T2, 48°C for 5 seconds; and T3, 47°C for 20 seconds. Subjective pain intensity was rated verbally (0–10), and pupil size was recorded continuously via infrared pupillometry. Analgesic effects for pain ratings and pupil sizes were quantified by comparing T2 maxima with T3 minima and analyzed using linear mixed-effects models.

**Results:**

Participants showed a strong OA effect, with pain ratings decreasing by 52% (*P* < 0.001, Cohen d = 2.09). Pupil sizes decreased by 20% (*P* < 0.001, Cohen d = 1.52), exceeding natural resting-state variability. Pain sensitivity measures were not associated with the amount of pupil size changes during OA.

**Conclusion:**

Pupil size decreased significantly during OA, consistent with the expected analgesic effect. Whether this truly reflects the endogenous pain modulatory system remains to be confirmed in future studies that include a constant-temperature control condition to establish specificity of the observed pupil response.

## Abstract 2

Supplemental Digital Content is Available in the Text.

Pupillometry might capture offset analgesia–like effects in healthy participants, providing a complementary physiological marker of endogenous pain modulation and potential tool to detect dysfunction in chronic pain.

## 1. Introduction

Offset analgesia (OA) is a common physiological phenomenon thought to reflect aspects of the endogenous pain modulatory system, as it has been associated with the activation of brain regions involved in descending pain modulation, including brainstem and prefrontal areas.^12,15,36^ Offset analgesia is described as a disproportionately large reduction in pain perception after a small decrease in stimulus intensity,^19^ commonly assessed through subjective pain ratings.

Psychological, emotional, and cognitive factors contribute to interindividual differences in pain perception, although findings across studies are not always consistent.^13,34,37,51,54,56^ These influences can bias self-reported pain ratings and complicate comparisons of OA across individuals. Therefore, physiological measures might reduce such bias and offer a more objective assessment of the pain modulatory system.

In this regard, the close anatomical and functional links between the pain and the autonomic nervous system make autonomic responses, promising physiological indicators of pain processing and modulation.^4,14,49^ For example, skin conductance and heart rate have frequently been used to measure autonomic activation during pain processing. These measures typically increase with stimulus intensity but do not necessarily reflect the perception of pain.^7,24,38^

Pupillometry, traditionally used in psychological and cognitive research, provides a noninvasive measure of autonomic correlates of pain states. Experimental work in acute pain studies showed that pupil dilation increases with stimulus intensity^10,11^ and is modulated by pain expectancy.^16,18^ Pupil size changes are also amplified in experimental models mimicking mechanisms of chronic pain,^8^ suggesting sensitivity to prolonged nociceptive states, although data on pupil size behavior in clinical chronic pain populations remain limited. Extending beyond an experimental context, pupillometry has further been applied in clinical and perioperative settings to objectively monitor pain states, which is particularly valuable in children, nonverbal, or critically ill patients.^41,57^

More recently, analgesia-related alterations in pupil size have been observed using a conditioned pain modulation (CPM) paradigm,^23^ which is commonly used to assess the function of the aforementioned endogenous pain modulatory system.^43,58^ Conditioned pain modulation is based on the concept of “pain inhibits pain,” where a conditioning stimulus reduces the perception of a painful test stimulus applied to a different body location, which reflects a spatial filtering mechanism.^43^ During OA, heat is applied at the same location throughout the paradigm and is therefore considered a temporal filtering mechanism.^20,30,61^

In this study, we investigated pupil size changes during OA to explore their potential as a physiological marker of the endogenous pain modulatory system to complement subjective pain ratings. We hypothesized that pupil size would reflect the analgesic effect caused by the paradigm. In addition, we assessed individual pain sensitivity using heat pain threshold and tolerance levels to explore whether baseline differences in nociceptive sensitivity relates to the magnitude of pupil size changes during OA.

## 2. Methods

### 2.1. Participants

Healthy female and male adults aged 18 to 40 years were recruited for this study. Exclusion criteria included acute pain or history of chronic pain, current use of pain or pupil-influencing medications (eg, H1-antihistamines), pregnancy, recent head injury, neurological disorders, depression, and prior eye injury/surgery. All participants provided written informed consent before participation. The study protocol was approved by the local ethics board (Kantonale Ethikkommission Zürich, 2024-00134) and conducted in accordance with the Declaration of Helsinki.

### 2.2. Study design

This prospective observational study comprised a main experiment and a subexperiment. The main experiment investigated pupil size changes during an OA paradigm. The subexperiment examined potential confounds in pupil size changes introduced by modifications to the OA paradigm, including auditory tone and verbal ratings instead of continuous visual analogue scale (VAS) (for details see below: 2.3.2 OA).

### 2.3. Main experiment

All measurements were conducted in a quiet, temperature-controlled room under constant lighting (220–250 lux; DEM300, Velleman, Belgium). Sensory integrity of the tested area, ie, left volar forearm was assessed using light touch, pin prick, vibration detection, and thermal sensation (warm and cold; Rolltemp II, Somedic SenseLab AB, Sweden). Heat pain threshold and tolerance were determined as a measure of pain sensitivity. Participants were then seated in a comfortable chair with their heads stabilized using a chin and forehead rest. They were instructed to focus on a black fixation cross displayed on a computer screen positioned in front of them. Pupil size was recorded using an infrared camera positioned between the screen and headrest. The thermode was placed on the left volar forearm, near the cubital fossa (inner elbow). Participants were informed that the thermode would change temperature and that the sensation might become uncomfortable. They were instructed to rate their current pain as quickly as possible upon hearing a tone. Baseline pupil size was measured for 5 seconds before trial onset. Each participant completed 1 OA trial.

#### 2.3.1. Heat pain threshold and tolerance

The heat pain threshold was determined using an Advanced Thermal Stimulation thermode (30 × 30 mm, Pathway Model, Medoc, Israel). The thermode was placed on the right volar forearm contralateral to the OA paradigm. Baseline thermode temperature started at 32°C and increased at a rate of 1°C/s. Participants were instructed to press a button on a computer mouse as soon as they felt an additional quality adding up to the sensation of warmth (eg, burning, stinging, drilling, pulling),^44^ to terminate the measurement.

Heat pain tolerance was assessed using the thermode placed on the right volar forearm, slightly shifted from the site used for threshold testing to avoid sensitization. Thermode temperature started at 32°C and increased at a slower rate of 0.3°C/s,^59^ to ensure that the maximum temperature (ie, 50°C) was not reached too quickly to avoid a ceiling effect. Participants were instructed to press a button when the heat became intolerable (ie, when they would withdraw their arm from the stimulus).

Measures for heat pain threshold and tolerance were repeated 3 times, and the mean values were used for further analyses.

#### 2.3.2. Offset analgesia

Continuous heat was applied using the Advanced Thermal Stimulation thermode. The protocol for OA followed established protocols^1,19^ and began at a baseline temperature of 35°C (T0) and increased to 47°C (T1) at a rate of 1.5°C/s. This temperature was maintained for 5 seconds, then elevated to 48°C (T2) for 5 seconds, followed by a reduction back to 47°C (T3) (cooling rate of 1.5°C/s) for an additional 20 seconds before the paradigm went back to baseline.^1,19,61^

The use of continuous pain ratings through a VAS, commonly implemented in previous studies,^1,19,28,30,61^ was not feasible during pupillometry, as it would have compromised our primary readout due to the shift in visual attention and unwanted eye movements. Participants were instructed to maintain a stable gaze at a fixation cross to ensure accurate pupil size recordings. Therefore, we adapted the OA paradigm and participants were asked to provide verbal pain ratings on a numeric rating scale (NRS) from 0 (no pain) to 10 (worst imaginable pain) at predefined time points throughout the paradigm. A brief auditory tone signaled each rating. The first tone occurred when the temperature first reached 47°C (T1) and was repeated every 5 seconds until the end of the paradigm. A schematic representation of the experimental paradigm is shown in Figure 1.

**Figure 1.**
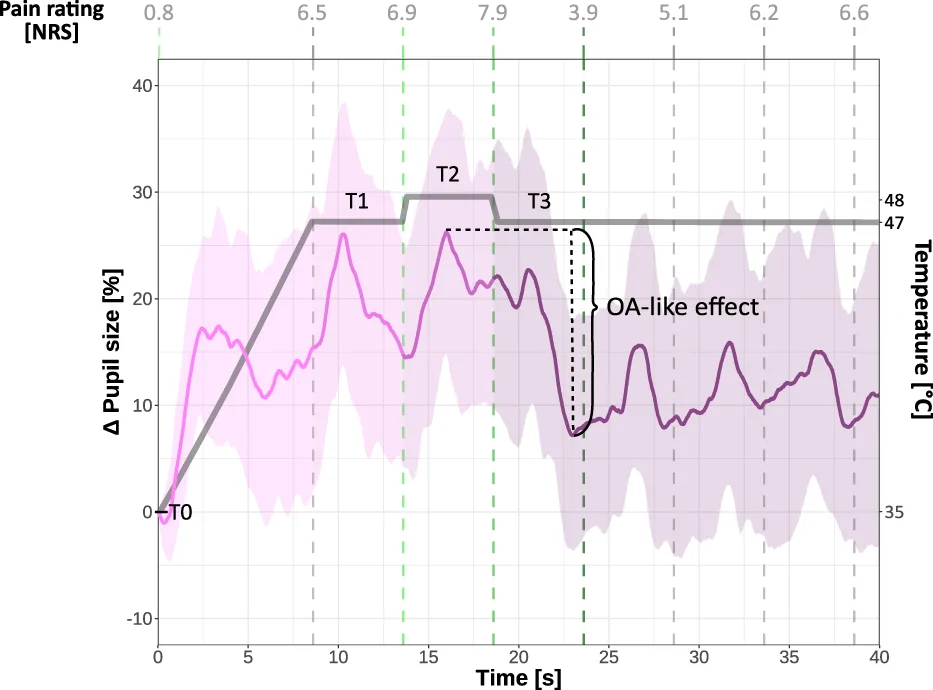
Continuous pupil size change during OA paradigm. The average pupil size changes relative to baseline (%) are shown throughout the OA paradigm (pink line) with shaded areas representing the SD. Different shades of pink indicate different time windows: T1 (47°C), T2 (48°C) and T3 (47°C) each lasting 5 seconds. The solid grey line represents the applied temperature profile. Vertical grey dashed lines indicate the timing of auditory cues and verbal pain ratings. Average pain ratings at the respective timepoints are displayed above the plot. Green vertical dashed lines highlight the timepoints from which individual pain ratings were extracted for statistical analysis. The black dashed line points out maximum and minimum changes in pupil size, indicating the magnitude of the OA-like effect. NRS, numerical rating scale; OA, offset analgesia.

The OA effect was calculated as the change from the maximum pain rating at 48°C (T2) to the minimum pain rating following the temperature reduction to 47°C (T3).^1,53^ The maximum pain rating was taken at the end of the 48°C phase (T2max), immediately before the temperature decrease. The minimum pain rating was taken 5 seconds after (T3min). The OA effect was expressed as percentage change according to this formula (Equation 1):

(1) $$OA\;effect=\frac{\left(T2\max-T3\min\right)}{T2\max}\;*\;100.$$

Higher values indicate a stronger analgesic response, suggesting a greater activation of the endogenous pain modulatory system.

#### 2.3.3. Pupillometry: data acquisition and processing

Binocular pupillometry was performed using the EyeLink 1000 plus infrared eye-tracking system (SR Research, Ontario, Canada) at 1000 Hz. Participants had normal or corrected-to-normal vision and were seated at a fixed distance of 90 cm from a computer screen (resolution: 1920 × 1080 pixels, maximum brightness of 250 cd/m^2^) where a black fixation cross was displayed. A chin and forehead rest was used to minimize head movements. A 9-point calibration, validation, and drift correction were performed. Pupil area was recorded in arbitrary units (measurement error <1%; <0.2% for sizes ≥ 3 mm).^50^ Baseline pupil size was averaged over 5 seconds before the OA paradigm. Continuous pupil size measures were recorded throughout the OA paradigm. Preliminary analysis revealed no significant differences in pupil size between the eyes. Therefore, subsequent analyses were conducted on the left eye only.

Pupil size data were downsampled to 100 Hz and preprocessed using a custom R script based on the *PupilPre* package. Blinks were excluded with a 150-millisecond (ms) padding window. Artifacts were detected using median absolute deviation and Mahalanobis distance thresholds, with a 200-ms padding window. Consecutive runs of extreme values or missing data were flagged, missing points linearly interpolated, and the data low-pass filtered (25 Hz). Raw data were converted to millimeters using the formula provided by SR Research.^49^

Baseline measures were used to compute coefficient of variation (CoV) for resting-state fluctuations, estimating relative pupil size variability.^3,47^ All pupil responses in the main experiment were baseline corrected^33^ and expressed as percentage change relative to baseline.

The primary analysis focused on maximum and minimum pupil size changes during OA. The decrease in pupil size, here referred to as the “OA-like effect,” was calculated by comparing the maximum pupil size at T2 (48°C) with the minimum in the first 5 seconds after the temperature decrease (T3, 47°C). The analysis of the minimum pupil size was restricted to this time window to avoid potential confounding effects from the subsequent tone presentation and pain rating. The OA-like effect on pupil size was quantified using the same formula applied to the pain ratings described above (Equation 1).

A supplementary analysis was conducted to confirm that primary findings were not dependent on extreme pupil size values. Mean percentage change relative to a 5-second baseline window was calculated within each time window (T1–T3).

In the subexperiment, stimulus-related pupil changes (tone and verbal rating) were baseline corrected, using the pupil diameter at T0 and expressed as absolute change in mm.

### 2.4. Subexperiment

We used a modified OA paradigm during pupillometry that included auditory tones followed by verbal pain ratings instead of a continuous VAS. Both auditory tones^26,27^ and verbal responding^9^ can induce pupil dilation, which may have influenced pupil size changes during the OA, potentially biasing the observed OA-like effect. Specifically, the maximum pupil size at T2 might be overestimated and/or the minimum pupil size at T3 might be underestimated. Therefore, a subexperiment was conducted 4 months later within a subset of participants. Environmental conditions and equipment were identical to the main experiment. The subexperiment used pupillometry to investigate additional effects of tone and rating. Measurements included 2 distinct conditions: (1) the main OA paradigm including heat, tone, and ratings and (2) heat stimulation only.

In (1), the same OA paradigm as in the main experiment was used. Participants fixated on a central cross and tone was presented every 5 seconds. At each tone, participants verbally rated their pain on an NRS 0 to 10.

In (2), heat stimulation was applied to the left volar forearm using the same paradigm described above. No auditory tones were presented, and participants did not provide verbal pain ratings. They were instructed to focus on the fixation cross until the trial ended. The 2 conditions were applied in a randomized order with a 5-minute break in between.

For each participant, the maximum pupil size change during T2 and the minimum pupil size change during T3, relative to the baseline, were extracted and compared between the conditions.

### 2.5. Statistical analyses

Statistical analyses were performed using R (version 4.5.1). Normal distribution of all data was tested by Shapiro-Wilk tests and histograms. Fulfillment of model criteria were checked with quantile-quantile plots and histograms. The statistical significance level was set to α = 0.05. All *P* values were adjusted for multiple comparisons using the Benjamini-Hochberg method. The effect sizes for OA (NRS) and OA-like effects (pupil size) were calculated using Cohen d for paired samples.

#### 2.5.1. Main experiment

Pain ratings and changes in pupil size across different time points (T0–T3 for pain ratings; T1–T3 for pupil size changes) were analyzed using general linear mixed-effects models. Pain ratings and pupil sizes changes in percentage relative to baseline served as dependent variables, time point as independent variable and participant as a random effect. Linear mixed-effects models were implemented using the *lme4* package in R. Post hoc pairwise comparisons between time points were conducted using estimated marginal means from the *emmeans* package. Pearson correlation was performed to assess the relationship between the OA effect in pain ratings and OA-like effect in pupil size. In addition, Pearson correlation analyses were performed between the OA-like effect and heat pain threshold and tolerance levels, to explore associations between pain sensitivity and the OA-like effect.

#### 2.5.2. Subexperiment

Differences in pupil responses between the subexperiment conditions (heat, tone and ratings vs heat stimulation only) were tested with paired *t*-tests in R using the *t.test* function from the *base stats* package. Specifically focusing on pupil size responses at T2 and T3. Offset analgesia-like effects were calculated from the mean pupil size data for both conditions and subsequently compared between conditions using a paired *t* test.

## 3. Results

### 3.1. Main experiment: pupil size changes during offset analgesia

#### 3.1.1. Participant characteristics

Thirty healthy participants were recruited for this study. One participant was excluded from the analysis due to intolerance of the heat stimulation during the OA paradigm. The remaining 29 participants demonstrated intact somatosensory function based on initial sensory bedside testing. The participant characteristics are presented in Table 1.

**Table 1:** Participant characteristics.

Sample size (n)	29
Age (y)	26.8 ± 3.8
Sex (male, female)	15, 14
Heat pain threshold (°C)	42.0 ± 2.2
Heat pain tolerance (°C)	47.2 ± 1.1

Data are shown as mean ± SD.

#### 3.1.2. Offset analgesia: pain ratings and pupillometry

Baseline pupil size, measured in a 5-second time window before the OA paradigm, showed an average pupil diameter of 3.17 ± 0.43 mm. The mean CoV in pupil size was 4.75%, reflecting normal fluctuations under resting state conditions.^2,29^ The average of the continuous pupil size changes relative to baseline and pain ratings during the OA paradigm are shown in Figure 1.

Linear mixed-effects models showed significant main effect of time point (T0–T3 pain ratings; T1–T3 pupil size change) for both pain ratings (F(3, 84) = 173.03, *P* < 0.001) and pupil size changes (F(2, 56) = 238.50, *P* < 0.001). Post hoc pairwise comparisons are reported in Supplementary Table S1 (https://links.lww.com/PR9/A452). In general, participants exhibited a robust analgesic effect, with pain ratings decreasing from a mean NRS of 7.9 ± 1.5 at T2 to 3.9 ± 2.2 at T3, corresponding to a 52% reduction in experienced pain (Cohen d = 2.09, *P* < 0.001, Fig. 2A). Similarly, pupil size decreased from 3.95 ± 0.54 mm at T2 to 3.16 ± 0.47 mm at T3, representing a reduction of 20% (Cohen d = 1.52, *P* < 0.001) exceeding the resting-state CoV, ie, 4.75%. When expressed relative to baseline, pupil size changes were 28.94 ± 11.92% at T1, 29.78 ± 12.54% at T2, and 3.47% ± 10.04% at T3 (Fig. 2B). Results of the secondary analysis also revealed a significant main effect of time point for pupil size changes (F(2, 56) = 11.12, *P* < 0.0001). For more detailed overview, see Supplementary Table S2 (https://links.lww.com/PR9/A452).

**Figure 2.**
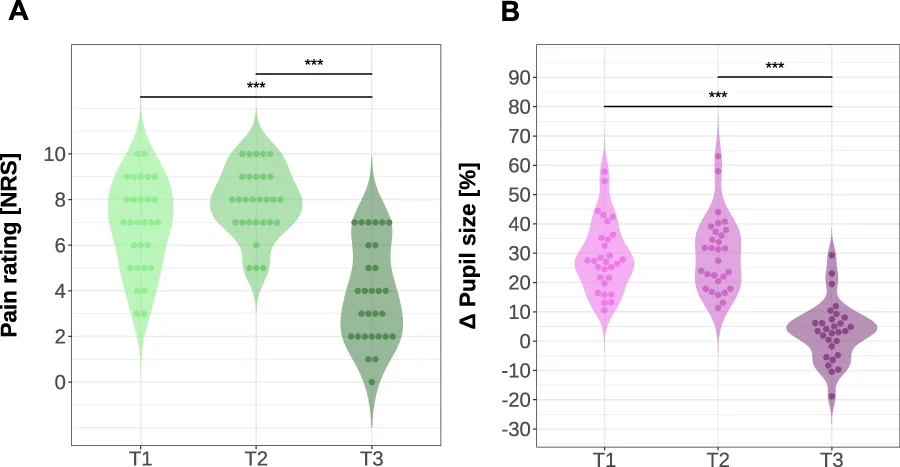
Pupil size reflects offset analgesia (OA). Boxplots with individual datapoints are shown for (A) pain ratings and (B) pupil size changes relative to baseline (%), across 3 time points during the OA-paradigm: T1 (47°C), T2 (48°C), and T3 (return to 47°C). ***P < 0.001. NRS, numerical rating scale.

Correlation analysis revealed that a higher OA effect in pain ratings was not associated with a higher OA-like effect in pupil size (r = −0.32, *P* = 0.09) (Fig. 3). Moreover, the OA-like effect was not associated with pain sensitivity measures (heat pain threshold: r = 0.18, *P* = 0.34; heat pain tolerance: r = −0.31, *P* = 0.10).

**Figure 3.**
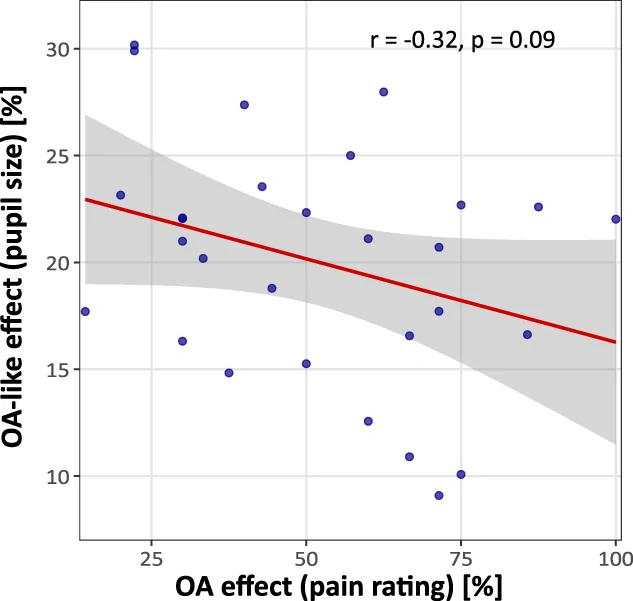
Association between OA (pain rating) and OA-like effect (pupil size). OA, offset analgesia.

### 3.2. Subexperiment: impact of auditory tone and verbal pain ratings on pupil size during offset analgesia

Six participants of the main experiment were included in the subexperiment and completed 2 experimental conditions, ie, (1) heat, tone, and rating and (2) heat stimulation only (Fig. 4). Paired *t*-tests of pupil size changes within T2 (t(5) = −0.63, *P* = 0.56) and T3 (t(5) = −0.42, *P* = 0.69) revealed no significant differences between the 2 conditions. Additionally, the calculated OA-like effect presented in both conditions (for (1) = 18.65 ± 4.97%, for (2) = 19.29 ± 9.04%) did not differ from each other (t(5) = 0.24, *P* = 0.82) and were comparable to the OA-like effect of the main experiment (20% pupil size decrease).

**Figure 4.**
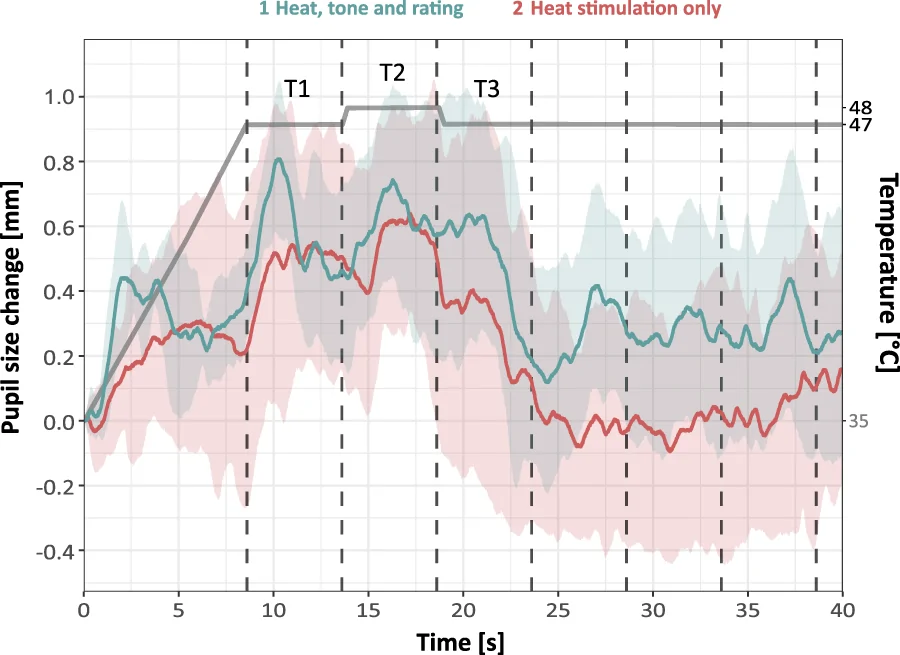
Pupil size change during the adapted offset analgesia (OA) paradigm and the heat only condition. Pupil size changes over a 40-second interval are shown for 2 experimental conditions: (1) OA paradigm with heat, tone, and verbal pain ratings (blue) and (2) OA paradigm with heat stimulation only (red). Vertical black dashed lines indicate the presentation of tone and subsequent verbal rating for condition (1).

## 4. Discussion

This is the first study examining changes in pupil size and pain ratings during an OA paradigm in healthy participants to determine whether the characteristic decrease in pain ratings is paralleled by a similar decrease in pupil size. Both measures showed significant reductions during OA. This pattern is consistent with an analgesic response, but whether pupil size changes specifically reflect endogenous pain modulation remains unclear. The magnitudes of these effects did not correlate, suggesting that pain ratings and pupil responses capture partially distinct aspects of the endogenous pain modulatory system. Together, these measures may provide complementary information with only partial overlap. In addition, pain sensitivity, ie, heat pain threshold and tolerance, were not associated with OA-like effects in this sample.

### 4.1. Main experiment: pain ratings and pupil size changes during offset analgesia

A recent study applied pupillometry during a CPM paradigm and reported that pupil size significantly decreased during the paradigm similar to the subjective experience of the participants.^23^ Comparable evidence for OA is currently lacking.

The present study observed a similar decrease in pupil size during OA emphasizing that pupillometry may be sensitive to endogenous pain modulatory processes across different paradigms. This is noteworthy because CPM and OA rely on distinct mechanisms (spatial vs temporal filtering),^20,61^ yet OA provides a simpler experimental setup and might be easier to implement.

Despite strong decreases in both verbal pain ratings and pupil size, the magnitudes of these OA effects were not correlated. This dissociation is in line with the study by Krafthöfer et al.^23^ investigating pupillometry during a CPM paradigm and suggesting that although both measures underlie top-down modulatory control, they are influenced by partially distinct neural mechanisms.

Offset analgesia represents the activity of the endogenous pain modulatory system, that involves the periaqueductal gray and rostroventromedial medulla, which primarily act on dorsal horn neurons to, in this case, inhibit nociceptive transmission to supraspinal centers.^39,40^ This reduction in nociceptive input contributes to decreased pain ratings during OA. However, subjective pain perception is not solely determined by nociceptive input. Pain ratings additionally reflect cortical integration of memory, attention, expectancy, and evaluative processes, involving somatosensory cortices, insula, anterior cingulate cortex, and prefrontal regions.^5,22,56^ By contrast, pupil size appears to depend more directly on activity of the locus coeruleus, which is coactivated by the periaqueductal gray and rostroventromedial medulla during noxious stimulation. The locus coeruleus modulates pupil size through sympathetic projections to the intermediolateral cell column and, together with hypothalamic inputs, controls autonomic outflow to the eye.^32,45,52^ Consequently, although both measures are influenced by descending modulatory circuits, their responses may diverge, as pupil size seems to reflect primarily brainstem-mediated arousal and stimulus saliency, whereas pain ratings are additionally shaped by cortical evaluative processes, including expectation, memory, and attention.^21^ This dissociation is further supported by pharmacological evidence. Previous studies have shown that agents affecting autonomic nervous system activity, such as decreasing heart rate and increasing heart rate variability, did not alter the magnitude of OA.^35,42^ Although OA is predominantly considered a centrally mediated phenomenon driven primarily by brainstem pain modulatory circuitry,^15,60^ peripheral contributions have not been fully excluded, as OA has been shown to depend on stimulus application sites^28^ and has proven largely resistant to pharmacological modulation.^25^ Although pupillometry cannot resolve the question of peripheral vs central mediation, it may provide an objective measure of brainstem-mediated activity, reflecting central processes that are engaged during OA, beyond what subjective pain ratings alone can capture.

The average OA effect was 52% in this study, which is lower than typically reported when pain is recorded continuously with a VAS (up to almost 100% decrease).^31,53^ Our discrete timing for pain ratings likely resulted in missing the exact time point of maximum analgesia, which may have led to a modest underestimation of the true OA effect. Likewise, the OA-like effect observed in pupil size may have been underestimated by the preset 5-seconds time windows.

#### 4.1.1. Effects of pain sensitivity measures on pupil size changes

The observed decrease in pupil size during OA was not associated with the participants' pain sensitivity (heat pain threshold and tolerance). Thus, greater thermal pain sensitivity did not correspond to a reduced modulatory function as reflected in pupil responses. Evidence directly linking pain threshold or tolerance measures to pupillary responses is lacking. Our analyses were therefore exploratory, testing whether individual differences in pain sensitivity might modulate the pupillary response, as some studies report correlations between pupil size and subjective pain intensity ratings under well-controlled, low cognitive-load conditions.^11,16^ We did not observe such associations in our data. Methodological differences, including the use of a modulation paradigm and additional cognitive or contextual influences, might explain this discrepancy.

Alternatively, the limited variability in pain sensitivity measures in our healthy cohort may have restricted the detection of potential relationships. Investigating these relationships in clinical pain cohorts with higher pain sensitivity might become more interesting in future studies.

### 4.2. Subexperiment: effect of confounding factors during the offset analgesia paradigm

The subexperiment was designed to assess whether auditory tones and verbal pain ratings influenced pupil responses during the OA paradigm. Pupil size is primarily controlled by ambient light conditions but can also vary under constant illumination due to cognitive and autonomic influences, including arousal,^6,17,32,46,52^ working memory,^2^ and speech production,^9^ as well as external sensory stimuli such as auditory input.^26,27^ These factors can modulate pupil size independently of nociceptive input and therefore may have confounded the interpretation of pupil size changes during the OA-paradigm. Comparing the 2 conditions, (1) heat with tone and ratings vs (2) heat alone, revealed no significant differences in maximum pupil size change at T2, minimum change at T3, or the calculated OA-like effect. These results suggest that the presence of auditory cues and the act of providing verbal ratings did not substantially alter pupillary responses in our paradigm, although the small sample size might restrict the strength of this conclusion. These results should therefore be considered preliminary and further studies with larger samples are needed to more conclusively determine the extent to which task-related factors may affect pupil responses in the OA paradigm.

### 4.3. Limitations

Although our findings suggest that pupils might reflect analgesic effects induced by the OA paradigm, several limitations should be considered. As this study introduces a novel physiological measure of OA, the absence of a dedicated control condition such as a constant-temperature trial limits the ability to fully exclude alternative explanations, such as time-dependent adaptation of the pupil response. Notably, all participants demonstrated a reduction in pupil size (−0.79 ± 0.24 mm), suggesting that the paradigm consistently induced the OA-like effect across participants. Nevertheless, future studies should include appropriate control conditions to strengthen the interpretation of our findings.

In the present study, we deliberately limited repeated applications of the paradigm to keep participants naive and to minimize potential influences of expectation. However, future studies should employ multiple OA trials to enhance measurement robustness and reduce pupil size variability.

## 5. Conclusion

The present study observed pupil size changes during an OA paradigm, which was consistent with the decrease in pain ratings typically seen during OA. Whether these pupil size changes reflect activation of the endogenous pain modulatory system remains to be determined by inclusion of control conditions in future studies. The absence of a correlation between changes in pupil size and pain ratings suggests that these measures may capture partially distinct aspects of pain processing. These results are preliminary, yet they offer a first indication that pupillometry may be sensitive to processes underlying OA and warrant further investigation.

## Disclosures

The authors have no conflicts of interest to declare.

## Supplemental digital content

Supplemental digital content associated with this article can be found online at https://links.lww.com/PR9/A452.
